# Clinical Characteristics and Predictors of Mortality in Elderly Patients Hospitalized with COVID-19 in Bangladesh: A Multicenter, Retrospective Study

**DOI:** 10.1155/2022/5904332

**Published:** 2022-06-11

**Authors:** Md. Asaduzzaman, Z. H. M. Nazmul Alam, Mohammad Zabed Jillul Bari, M. M. Jahangir Alam, Shishir Ranjan Chakraborty, Tasnim Ferdousi

**Affiliations:** ^1^Department of Medicine, Sylhet MAG Osmani Medical College Hospital, Sylhet 3100, Bangladesh; ^2^Department of Medicine, Sylhet MAG Osmani Medical College, Sylhet 3100, Bangladesh; ^3^Department of Ophthalmology, Bangabandhu Sheikh Mujib Medical University, Dhaka, Bangladesh

## Abstract

**Purpose:**

Elderly patients are at high risk of fatality from COVID-19. The present work aims to describe the clinical characteristics of elderly inpatients with COVID-19 and identify the predictors of in-hospital mortality at admission.

**Materials and Methods:**

In this retrospective, multicenter cohort study, we included elderly COVID-19 inpatients (*n* = 245) from four hospitals in Sylhet, Bangladesh, who had been discharged between October 2020 and February 2021. Demographic, clinical, and laboratory data were extracted from hospital records and compared between survivors and nonsurvivors. We used univariable and multivariable logistic regression analysis to explore the risk factors associated with in-hospital death. *Principal Results*. Of the included patients, 202 (82.44%) were discharged and 43 (17.55%) died in hospital. Except hypertension, other comorbidities like diabetes, chronic kidney disease, ischemic heart disease, and chronic obstructive pulmonary disease were more prevalent in nonsurvivors. Nonsurvivors had a higher prevalence of leukocytosis (51.2 versus 30.7; *p*=0.01), lymphopenia (72.1 versus 55; *p*=0.05), and thrombocytopenia (20.9 versus 9.9; *p*=0.07). Multivariable regression analysis showed an increasing odds ratio of in-hospital death associated with older age (odds ratio 1.05, 95% CI 1.01–1.10, per year increase; *p*=0.009), thrombocytopenia (OR = 3.56; 95% CI 1.22–10.33, *p*=0.019), and admission SpO_2_ (OR 0.91, 95% CI 0.88–0.95; *p*=0.001).

**Conclusions:**

Higher age, thrombocytopenia, and lower initial level of SpO_2_ at admission are predictors of in-hospital mortality in elderly patients with COVID-19.

## 1. Background

Since December 2019, countries all over the world have been confronted with an unprecedented challenge, a battle against the severe acute respiratory syndrome coronavirus (SARS-CoV-2), with a high fatality rate. In the majority of patients, coronavirus disease 2019 (COVID-19) causes only mild-to-moderate illness with respiratory and flu-like symptoms [1]. However, it has been reported to be severe and critical in 14% and 5% of patients, respectively, and requires intensive care support with mechanical ventilation [2]. The mortality in the critical group of patients is high [3]. Since the beginning of the SARS-CoV-2 outbreak, it was evident that older people, compared to younger ones, were at higher risk of getting the infection and developing more severe diseases with unfavorable prognosis [1, 4, 5]. Data from China and Italy suggest a case fatality of 2.3% in patients with COVID-19. Case fatalities in Italy appear to be in the elderly age groups of 60 and above, whereas more than 50% of the fatalities in China are in ages greater than 50 [6]. However, the reasons why older people are at significantly increased risk of severe disease following infection from COVID-19 are not clear. Compared to younger patients, a lesser percentage of elderly patients manifest the classical triad of the disease (fever, cough, and dyspnea) this makes an earlier diagnosis of COVID-19 in these patients difficult and delayed, which may contribute to increased mortality [7, 8] Importantly, higher prevalence of comorbidity which is linked to severe disease course and poor prognosis may pose elderly at more risk than younger group [9]. Moreover, immunosenescence and malnutrition can synergistically contribute to the augmented susceptibility and worse outcome of aged people to SARS-CoV-2 [10].

Detection of risk factors for mortality is an important component of the strategies for managing COVID-19. This information is more important at a time when the demand for critical care is upsurging, and the resources for healthcare are limited. Keeping this in mind, we aim to identify the risk factors for in-hospital mortality at admission in the elderly COVID-19 patients. We considered clinical aspects, presence of comorbidities, and laboratory parameters as well as in-hospital outcomes.

## 2. Subjects, Materials, and Methods

### 2.1. Study Methods and Data Collection

**Figure 1:**
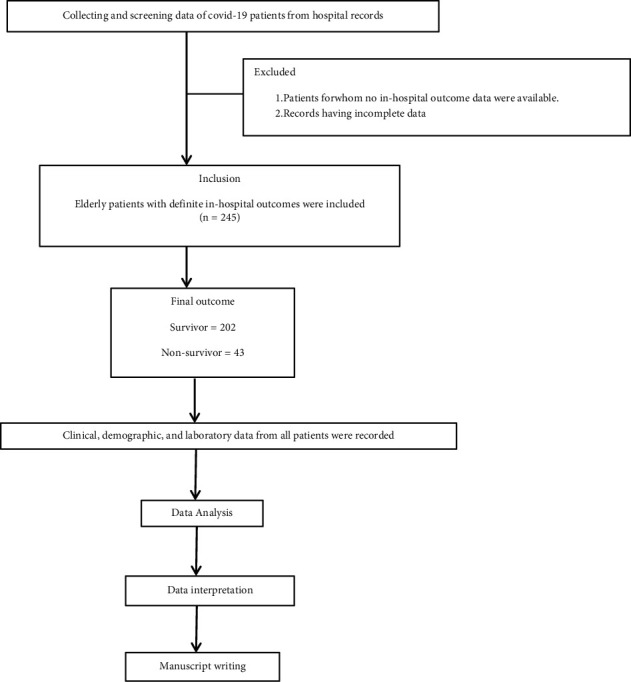
Flowchart of this present research process.

Data were extracted from the hospital records of elderly patients (age >60 years) who had been admitted with a diagnosis of COVID-19 in four COVID-19 designated hospitals in Sylhet, Bangladesh (a major city in north-eastern Bangladesh), during the pandemic crisis of severe acute respiratory syndrome coronavirus-2 (SARS-CoV-2) between October 2020 and February 2021. Those without a definitive outcome during their hospitalization were excluded from the study. Subjects with missing data in the records were also excluded. Clinical, demographic, and laboratory data from all patients were recorded. The clinical diagnosis of COVID-19 was made when patients met one of the two following criteria: (I) a positive RT-PCR for SARS-CoV-2 or (II) pulmonary abnormality characteristics of COVID-19 found on chest X-ray or chest CT scan based on the radiological criteria of COVID-9 infection. As flowchart of the research process is an important part of the scientific article [11], we have given it in Figure 1.

### 2.2. Study Variables

The outcome variable was in-hospital death (nonsurvivors and survivors), a binary variable. The demographic data included here are age, sex, and length of hospital stay in days (LOS). Clinical data included here are clinical features (fever, cough, respiratory distress, fatigability, loss of smell, diarrhea, sore throat, anorexia, and chest pain); the presence of comorbidities like hypertension, chronic kidney disease (CKD), chronic obstructive pulmonary disease (COPD), diabetes mellitus (DM), ischemic heart disease (IHD), cerebrovascular accident (CVA), and peripheral capillary oxygen saturation (SpO_2_) at admission; types of respiratory support required (not required supplemental oxygen (No O_2_), low flow (<4 liters/minute), high flow nasal cannula (HFNC), and noninvasive ventilation (NIV), and those required invasive mechanical ventilator support (ventilator)). Laboratory parameters included complete blood count (CBC), D-dimer, S. ferritin, and blood glucose (BG). The radiographic findings included are chest CT scan reports.

### 2.3. Statistical Analysis

Patients' demographic and clinical characteristics were analyzed using descriptive statistics. Continuous and categorical variables are expressed as medians (interquartile ranges) or mean (standard deviation (SD)) and as frequency (%), respectively. The Shapiro–Wilk test was used to assess the normality of continuous variables. We presented continuous measurements by the mean and standard deviation (SD) for data that followed a normal distribution and by the median and interquartile range (IQR) for data that were skewed. Patients were included in either the survivor or nonsurvivor group. The mean difference between two groups (survivor versus nonsurvivor) in a continuous variable was assessed using a two-independent-sample mean test (*t*-test) for the normally distributed data and using nonparametric Mann–Whitney *U* test for the nonnormally distributed data. The Chi-square test (Χ^2^ test) of independence was used to determine the association (difference) among categorical variables. A multiple logistic regression model was used to identify the risk factors for in-hospital death. The candidate predictors for the final model were selected based on clinical relevance and by performing standard model building procedures (backward selection and least AIC value). Initially, simple logistic regression models were fitted for each of the candidate predictors. The factors that were significantly associated with in-hospital death in the simple logistic regression models (*p* < 0.05) were included in the final multiple logistic regression model. The variables that were highly correlated or associated with each other were excluded from the model due to multicollinearity. Goodness of fit of the model was assessed using the Hosmer–Lemeshow test and area under the ROC curve (AUC). Model findings were presented using odds ratio (OR) and 95% confidence interval (CI). A *p* value <0.05 was considered statistically significant. Analysis was performed using *R* software. This study is reported following the STrengthening the Reporting of OBservational studies in Epidemiology (STROBE) [12] statements.

## 3. Results

### 3.1. Demographics and Baseline Characteristics of Patients

We included 245 inpatients in the final analysis. 43 (17.55%) patients died during hospitalization, and 202 were discharged following recovery. The mean age of all patients was 70 ± 8.3 years, while that of patients in the survivor group was 69.3 ± 7.9 years. Nonsurvivor had a higher mean age of 73.6 ± 9.5 years. Most patients (64.5%) were male. The most prevalent comorbidities were hypertension (79.6%), DM (71.4%), IHD (30.6%), CKD (22.9%), COPD (13.1), and CVA (7.3%). Compared to survivor, prevalence of DM (76.7% versus 70.3%; *p*=0.507), IHD (46.5% versus 27.2%; *p*=0.021), CKD (32.6% versus 20.8%; *p*=0.142), and COPD (25.6% versus 10.4%; *p*=0.015) was higher in nonsurvivors (Table 1).

The most common symptoms on admission were fever (90.2%) and cough (71.4%), followed by shortness of breath (SOB) (69.4%), fatiguability (56.7%), loss of smell (19.6%), diarrhea (15.9%), and sore throat (11%). The presence of sore throat, fever, SOB, fatiguability, and diarrhea was higher in the nonsurvivor group, whereas cough and loss of smell were more common in the survivor group (*p* < 0.001). Nonsurvivors had significantly lower SpO_2_ at admission than survivors (median; 84 versus 93; *p* < 0.001). Length of hospital stay was significantly higher in nonsurvivors (10.5 ± 5.7% versus 8.6 ± 4.3%; *p*=0.015) (Table 1).

Regarding respiratory support, a higher percentage of nonsurvivors required ventilator (37.2% versus 1.55; *p* < 0.001), noninvasive ventilation (NIV) (44.2% versus 1.5; *p* < 0.001), and high flow nasal cannula (HFNC) (16.3% versus 10.9; *p*=0.463) (Table 1).

### 3.2. Laboratory Findings

Table 2 summarizes the laboratory results of the study population. Median WBC count (10.2 versus 7.79; *p*=0.002) and neutrophil count (8.87 versus 5.85; *p* < 0.001) were significantly higher in the nonsurvivor group, while the lymphocyte count (1.04 versus 1.32; *p*=0.309) was lower. A higher proportion of patients in the nonsurvivor group had leukocytosis (51.2 versus 30.7; *p*=0.01), neutrophilia (86 versus 69.8; *p*=0.04), lymphocytopenia (72.1 versus 55; *p*=0.05), and thrombocytopenia (20.9 versus 9.9; *p*=0.07). Compared to survivors, nonsurvivors had higher levels of blood glucose (median, 12.1 versus 9.8; *p*=0.07), D-dimer (median, 900 versus 793; *p*=0.62), and ferritin (median 474 versus 332; *p*=0.96) at admission (Table 2).

Regarding radiographic changes, a higher percentage of patients had bilateral lung infiltrate in the nonsurvivor group (90.6% versus 83.7%; *p*=0.29). The most common change is ground-glass opacities (GGO) (63.3%), followed by the simultaneous presence of GGO and consolidation (GGO_Cons) (27.8%) and then consolidation alone (9%).

### 3.3. The Risk of In-Hospital Death

**Figure 2:**
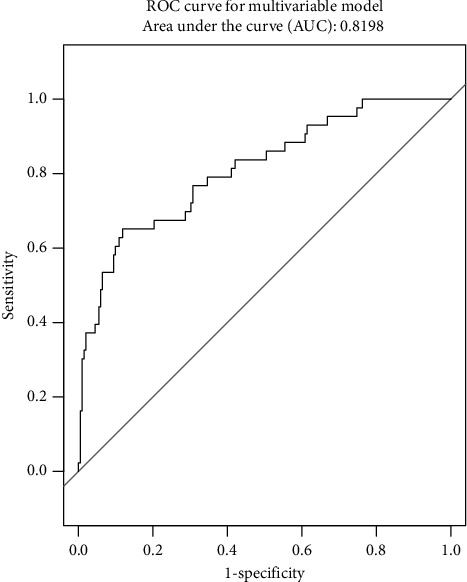
ROC curve for the multivariable model predicting mortality.

Table 3 shows the result of logistic regression analysis for risk factors for mortality of COVID-19 patients. The univariable analysis found higher odds of in-hospital death in patients with COPD and IHD. Age, WBC counts, neutrophil counts, leukocytosis, lymphocytopenia, thrombocytopenia, NLR, and SpO_2_ were also significantly associated with death (*p* < 0.05). The multivariate regression analysis showed that the risk of in-hospital death increased with age (odds ratio (OR): 1.05, 95% CI (confidence interval): 1.01–1.10,*p*=0.009), thrombocytopenia (aOR: 3.56, 95% CI: 1.22–10.33, *p*=0.01), and lower SpO_2_ (aOR: 0.91, 95% CI: 0.88–0.95, *p*=0.001). The area under the curve (AUC) of the multivariable model is 0.8198, which is considered excellent (Figure 2).

## 4. Discussion

### 4.1. Statement of Principal Findings

In this study, we summarized the clinical and laboratory characteristics of elderly patients (*n* = 245) diagnosed with COVID-19. We identified predictors of COVID-19-related deaths. Nonsurvivors were older and had a higher prevalence of comorbidities than survivors. A higher proportion of patients in the nonsurvivor group had leukocytosis, neutrophilia, lymphocytopenia, and thrombocytopenia as well as higher levels of blood glucose, D-dimer, and ferritin. Nonsurvivors were admitted with a more severe degree of hypoxemia than survivors (<0.001). Advanced respiratory support was required more frequently in nonsurvivors. After adjustment for potential covariates, higher age, thrombocytopenia, and lower initial SpO_2_ were found to be independently associated with in-hospital mortality.

### 4.2. Strengths and Limitations

Considering the vulnerability of older people to COVID-19, there is an urgent need to identify predictors of poor outcomes in this group of patients. The developing countries with their limited healthcare resources are badly affected by the challenges placed by the COVID-19. Prioritizing the resources to the vulnerable groups is one of the best options to address this issue and reduce the death toll. This study will contribute in this regard. Here, we studied demographic and clinical characteristics as well as commonly done and cost-effective hematological tests and analyzed their ability to predict the prognosis of patients. We collected data from four COVID-19 designated hospitals in Sylhet. We believe that our study sample is representative of hospitalized elderly patients in Bangladesh.

This study has some limitations. First, this is a retrospective study focused on hospitalized patients. Hospitalized patients usually present with severe disease and consequently have a higher mortality rate, and that is why our data may overestimate overall mortality in the entirety of the older patients with COVID-19. Second, some patients did not have laboratory data for some biochemical values, such as procalcitonin, LDH, lactate, and interleukin-6 serum levels, which may have led to an underestimation of their potential predictive value. Moreover, we could not collect any frailty scale data in this study. In geriatric patients, the level of frailty has been reported to be a useful predictor of short-term COVID-19 outcomes [13].

### 4.3. Interpretation in the Context of the Wider Literature

Most studies published so far have demonstrated a higher risk of worse outcomes following COVID-19 disease in elderly people. The higher the age, the higher the case fatality rate [14]. We found a case fatality rate in hospitalized patients ≥60 years old of age (17.5%) that was similar to a recent systematic review and meta-analysis [15] but higher than what was reported in China [16] and lower than that reported from a long-term care facility in Italy [17]. Regional variation in case fatality may be due to heterogeneity in testing and reporting system [18], difference in healthcare delivery system [19], genetic variability [20, 21], environmental factors [22], and notably virus strains [23]. But what makes elderly people more vulnerable to COVID-19 infection is not a clear/a matter of debate. Overexpression of ACE-2, immune alteration in the elderly, mitochondrial dysfunction, decreased physical activity, hormonal changes, and poor nutrition all may contribute to increased susceptibility to severe disease and death in the elderly [24].

The co-occurrence of chronic diseases in the elderly is increasingly becoming one of the most pressing public health concerns in most of the world. It was reported that more than half of the elderly in developed countries had more than three chronic diseases, meaning that an individual suffers from two or more diseases with different pathology and no mutual dependence at the same time [25, 26].

Regardless of ethnicities, the presence of comorbidities significantly increases the chance of contracting the disease and the risk of developing the severe disease with poor outcomes [27]. This is evidenced in both hospitalized patients [28–30] and a recent population-based cohort study [31]. Moreover, the elderly patient has a higher burden of comorbidities than nonelderly patients, which could explain the poor prognosis in this group of patients. This present study noticed a higher proportion of comorbidities, such as diabetes mellitus, ischemic heart disease, chronic kidney disease, and chronic obstructive pulmonary disease in nonsurvivors which is in good agreement with other studies [27, 32]. Hence, clinicians should treat them with more attention considering high-risk groups.

Though COVID-19 is a respiratory infection, it has a significant impact on the hematopoietic system and hemostasis. Hematologic consequences of this new infection have prompted the medical community to think about new treatment approaches. Changes in peripheral blood cell counts have been well-studied in COVID-19. The most notable features are an increase in the counts of white blood cells and neutrophils, whereas counts of lymphocyte and platelet decrease [33, 34]. In several recent meta-analyses, that included geriatric patients, low lymphocyte count, low platelet count, and high neutrophil count, were found to correlate significantly with mortality [33, 35, 36].

Our study found a lower lymphocyte count in nonsurvivors which is consistent with other studies [33, 34]. The possible mechanism of lymphocytopenia is the direct cytotoxic effect of the virus on lymphocytes as there is evidence that ACE-2 receptors are expressed on lymphocytes, direct damage of lymphatic organs, and inflammatory cytokines that continued to be disordered, perhaps leading to lymphocyte apoptosis [37].

Similar to existing studies [33–36], this study also observed a lower platelet count in nonsurvivors and identified thrombocytopenia as a risk factor for mortality in elderly COVID-19 patients. The potential reasons for thrombocytopenia include the direct effect of SARS-CoV-2 on platelet production, autoimmune destruction of platelets, or increased platelet consumption. Secondary hemophagocytic lymphohistiocytosis causes excessive proliferation and activation of macrophages and, in turn, produces a surge in inflammatory cytokines. It has been postulated that this cytokine storm damages hematopoietic progenitors and reduces platelet production [38, 39].

A systematic review and meta-analysis of 78 studies revealed that, increased total WBC count found on admission was a risk factor for mortality and a stepwise increase in risk for mortality in parallel with the increase of the total WBC threshold. Increased baseline absolute neutrophil count (ANC) was found to be a risk factor for intensive care requirements [40]. Consequently, a high neutrophil-to-lymphocyte ratio (NLR) on admission was associated with severe COVID-19 and mortality [41]. Neutrophilia may be due to COVID-19-associated immune dysregulation that leads to neutrophil production. Additionally, neutrophilia can be secondary to a superimposed bacterial infection, which is more likely to occur in patients with severe disease [42]. Consistent with these findings, our study also found a higher leukocyte and neutrophil count and a lower lymphocyte and platelet count in nonsurvivors. Also, a significantly higher value of NLR was observed in nonsurvivors of this study.

COVID-19 has been described as a thromboinflammatory syndrome. In patients who developed severe COVID-19, several conditions, including sepsis, complement activation, cytokine storm, endothelial damage, and inflammatory and microthrombotic pathway activation, predispose patients to thrombosis and coagulopathy. D-dimer is a well-known marker for evaluating thrombotic events. The frequency of D-dimer elevation has been reported to be 36–43% in COVID-19 patients [43]. Patients with elevated D-dimer levels had 1.58 times higher risk for progression to more severe clinical status [44]. In addition, it was also found that the D-dimer levels were higher in nonsurviving patients compared to surviving patients, and also patients with elevated D-dimer levels had a 1.82-fold higher risk for mortality compared to other patients [45]. Consistent with this, our study found a higher level of D-dimer in nonsurvivors when compared with survivors. But this study did not find D-dimer as a risk factor for mortality.

Ferritin, an acute phase reactant, may be a mediator of immune dysregulation in COVID-19 [46]. There is a complex interplay between ferritin and cytokines. The various suggested mechanisms of raised ferritin levels are the proinflammatory cytokines like IL-16 and TNF-*α* promoting synthesis of ferritin and leakage of intracellular ferritin by cellular damage [47]; on the other hand, ferritin can induce the expression of pro- and anti-inflammatory cytokines as well. In this regard, there are arguments in favor of adding COVID-19 to the spectrum of hyperferritinemic syndrome [48]. Numerous systematic reviews and meta-analyses observed a high ferritin level in severe disease, and ferritin was found to be a prognostic factor [49, 50]. But data from two Italian COVID-19 units demonstrated that ferritin levels over the 25th percentile were associated with a more severe pulmonary involvement, independently of age and gender, and not associated with disease outcomes [51]. This present study found a higher level of ferritin in patients who died but did not find it as a predictor of mortality which agrees with the Italian cohort [52].

There is a bidirectional relationship between COVID-19 and hyperglycemia. The high blood glucose level at admission is associated with severe disease and poor outcomes [52–54]. On the other hand, COVID-19 is associated with new-onset hyperglycemia or diabetes as well as worsening of preexisting diabetes [55, 56]. Possible mechanisms of hyperglycemia in COVID-19 are direct virus-mediated beta-cell damage, triggering of beta-cell autoimmunity by the virus, disorganized and exuberant immune response against the virus, which leads to perturbations in glycemic status, and iatrogenic hyperglycemia caused by corticosteroids [57]. Our study finding is also consistent with these and we found, compared to survivors, nonsurvivor had a higher blood glucose levels at admission.

Many patients, particularly the elderly who later develop respiratory failure, experience hypoxemia and hypocapnia without signs of respiratory distress. This is called “happy hypoxemia” or “silent hypoxemia.” Earlier, it was described in patients during the initial Wuhan outbreak [58], and a recent study found this silent hypoxemia as a poor prognostic marker in COVID-19 [59]. At admission, objective signs of respiratory compromise such as oxygen saturation and respiratory rate are associated with markedly elevated mortality [60]. This present study found a significantly lower SpO_2_ level at admission in nonsurvivors when compared with survivors. After adjusting for potential covariates, admission SpO_2_ was found to be an independent predictor of mortality.

This present study observed that a higher proportion of patients in the nonsurvivor group have bilateral involvement in chest imaging in comparison to the survivor. This finding is in good agreement with studies reported by Pan et al. [61] and Li et al. [62].

### 4.4. Implications for Policy, Practice, and Future Research

These pieces of evidence advocate for judicious decisions on resource allocation in overwhelmed healthcare systems. As per this study's findings concern, patient characteristics, including age, platelet count, and oxygen saturation status at admission, may be significant predictors of death in elderly patients with COVID-19. As COVID-19 is an evolving disease and its course is unpredictable, therefore, elderly patients need special attention and care. To curb the outbreak and reduce the pressures on the healthcare system, it is important that policy-makers must prioritize the high-risk group in their strategic planning. Further larger studies are necessary to better understand and confirm our findings, to rapidly identify characteristics associated with a poor outcome among elderly patients suffering from COVID-19 and provide better care.

## Figures and Tables

**Table 1:** Demographic and clinical characteristics of patients with COVID-19.

	Total, *n* = 245	Survivor, *n* = 202	Nonsurvivor, *n* = 43	*p* value
Age, mean (±SD)	70 ± 8.3	69.3 ± 7.9	73.6 ± 9.5	0.002

*Sex*				0.666
Male	158 (64.5%)	132 (65.3%)	26 (60.5%)	
Female	87 (35.5%)	70 (34.7%)	17 (39.5%)	

*Comorbidity*				
Hypertension	195 (79.6%)	162 (80.2%)	33 (76.7%)	0.763
DM	175 (71.4%)	142 (70.3%)	33 (76.7%)	0.507
IHD	75 (30.6%)	55 (27.2%)	20 (46.5%)	0.021
CKD	56 (22.9%)	42 (20.8%)	14 (32.6%)	0.142
COPD	32 (13.1%)	21 (10.4%)	11 (25.6%)	0.015
CVA	18 (7.3%)	14 (6.9%)	4 (9.3%)	0.826

*Clinical characteristics*				
Fever	221 (90.2%)	180 (89.1%)	41 (95.3%)	0.333
Cough	175 (71.4%)	145 (71.8%)	30 (69.8%)	0.937
SOB	170 (69.4%)	138 (68.3%)	32 (74.4%)	0.544
Fatiguability	139 (56.7%)	114 (56.4%)	25 (58.1%)	0.972
Loss of smell	48 (19.6%)	42 (20.8%)	6 (14%)	0.415
Diarrhea	39 (15.9%)	29 (14.4%)	10 (23.3%)	0.223
Sore throat	27 (11%)	16 (7.9%)	11 (25.6%)	0.002
Anorexia	4 (1.6%)	2 (1%)	2 (4.7%)	0.29
Chest pain	4 (1.6%)	4 (2%)	0 (0%)	0.789
LOS^*∗*^, median (IQR)	8.9 (4.6)	8.6 ± 4.3	10.5 ± 5.7	0.015
SpO_2_ at admission		93 (88–95.75)	84 (71–88.50)	<0.001

*Type of respiratory support required*				
Ventilator	19 (7.8%)	3 (1.5%)	16 (37.2%)	<0.001
NIV	22 (9%)	3 (1.5%)	19 (44.2%)	<0.001
HFNC	29 (11.8%)	22 (10.9%)	7 (16.3%)	0.463
Low flow oxygen	155 (63.3%)	154 (76.2%)	1 (2.3%)	<0.001
No O_2_	20 (8.2%)	20 (9.9%)	0 (0%)	0.065

DM, diabetes mellitus; IHD, ischemic heart disease; CKD, chronic kidney disease; COPD, chronic obstructive pulmonary disease; CVA, cerebrovascular accident; SpO_2_, peripheral capillary oxygen saturation; NIV, noninvasive ventilation; HFNC, high flow nasal cannula; No O_2_, no oxygen required. ^*∗*^LOS, length of stay.

**Table 2:** Radiology and laboratory findings (median (IQR)/number (%)).

Variables	Normal range	Total, *n* = 245	Survivor, *n* = 202	Nonsurvivor, *n* = 43	*p* value
TC WBC (× 10^9^/L)	4–10	8.2 (6–12)	7.79 (6–11.07)	10.2 (6.85–14.32)	0.002
>10		84 (34.3%)	62 (30.7%)	22 (51.2%)	0.017
<4		8 (3.3%)	8 (4%)	0 (0%)	0.393
Neutrophil (× 10^9^/L)	2.0–7.0	6.31 (4.24–9.82)	5.85 (4.15–9.40)	8.87 (4.82–13.44)	<0.001
>7 (× 10^9^/L)		178 (72.7%)	141 (69.8%)	37 (86%)	0.048
Lymphocyte (× 10^9^/L)	0.8–4.5	1.30 (.88–1.83)	1.32 (0.94–1.88)	1.04 (0.70–1.8)	0.309
<0.8		142 (58%)	111 (55%)	31 (72.1%)	0.058
NLR			4.42 (2.72–8.57)	7.08 (4.11–16.76)	<0.001
Platelet (× 10^9^/L)	150–350	220 (180–297)	232 (180–300)	209 (160–248)	0.133
<150		29 (11.8%)	20 (9.9%)	9 (20.9%)	0.076
>350		40 (16.3%)	32 (15.8%)	8 (18.6%)	0.827
D-dimer (ng/L)	0–500	821 (430–1706)	793 (420–1705)	900 (510–1811)	0.629
S. ferritin	20–300	353 (165–795)	332 (166–793.75)	474 (167.5–802)	0.961
Blood glucose	4.4–7.2	10.5 (8–14)	9.8 (8–14)	12.1 (9.4–14.35)	0.074

*Lung involvement on chest imaging*					
Unilateral infiltrate		26 (10.6%)	19 (9.4%)	7 (16.3%)	0.291
Bilateral infiltrate		219 (89.4%)	36 (83.7%)	183 (90.6%)	0.291

*HRCT findings*					
GGO		155 (63.3%)	135 (66.8%)	20 (46.5%)	0.02
Consolidation		22 (9%)	16 (7.9%)	6 (14%)	0.336
GGO + consolidation		68 (27.8%)	51 (25.2%)	17 (39.5%)	0.087

TC WBC, total count of white blood cells; NLR, neutrophil-to-lymphocyte ratio; GGO, ground-glass opacity.

**Table 3:** Predicting factors for death of the COVID-19 patients: logistic regression analysis.

Univariable				Multivariable^$^		
Variables	OR	95% CI	*p* value	aOR	95% CI	*p* value
Age	1.05	1.03–1.08	0.001	1.05	1.01–1.10	**0.009**
Male	0.81	0.41–1.61	0.586			
Female	1.23	0.61–2.41	0.544			
DM	1.39	0.66–3.14	0.397			
Hypertension	0.81	0.380–1.86	0.61			
CKD	1.83	0.87–3.74	0.0985			
COPD	2.96	1.27–6.64	0.009	1.43	0.54–3.75	0.463
IHD	2.32	1.17–4.56	0.0143	1.96	0.86–4.44	0.107
CVD	1.37	0.37–4.08	0.59			
WBC	2.70	1.34–5.47	0.00549	1.88	0.82–4.33	0.134
Leukocytosis	2.36	1.21–4.64	0.0116			
Neutrophil count	2.57	1.42–4.66	0.0017			
Lymphocyte count	0.65	0.38–1.11	0.119			
Lymphocytopenia	2.11	1.05–4.50	0.0416			
Platelet count	0.51	0.23–1.14	0.103			
Thrombocytopenia	2.40	0.97–5.61	0.0471	3.56	1.22–10.33	**0.01**
NLR	2.01	1.33–3.02	0.001			
D-dimer	1.07	0.79–1.44	0.6334			
Ferritin	1.05	0.78–1.42	0.70			
Blood glucose	2.35	0.98–5.60	0.05			
Admission SpO_2_	0.91	0.88–0.94	0.001	0.91	0.88–0.95	**0.001**

^$^Values that were significant in univariable analysis were included in the multivariable model. OR, odds ratio; aOR, adjusted odds ratio; CI, confidence interval.

## Data Availability

The datasets analyzed during the current study are not publicly available because of having no permission from the hospitals from where data were collected.
